# Dentin and Enamel Exposure on Upper Incisors for Bonding Buccal Laminated Veneers: Method Validation and Ex Vivo Quantification

**DOI:** 10.1155/ijod/9560887

**Published:** 2026-04-08

**Authors:** Constance Cuny, Manon Udar, Thibault Canceill, Markus B. Blatz, Antoine Galibourg

**Affiliations:** ^1^ Department of Dentistry – Oral Rehabilitation, Faculty of Health – University of Toulouse, Toulouse, France; ^2^ Department of Preventive and Restorative Sciences, University of Pennsylvania School of Dental Medicine, Philadelphia, Pennsylvania, USA, upenn.edu

**Keywords:** dental veneer, esthetic dentistry, minimally invasive dentistry, prosthodontics

## Abstract

**Objectives:**

This study compared the accuracy of postetching macrophotography, a clinically applicable method, with microscope observation (the laboratory reference) for quantifying enamel and dentin surfaces. A secondary objective was to assess the proportion of exposed dentin and enamel at different veneer preparation depths and to develop a classification system to optimize bonding protocols based on dentin exposure.

**Methods:**

Twenty‐five human maxillary incisors underwent three sequential buccal reduction steps corresponding to ceramic laminate veneer (LV) preparation: minimally invasive preparation (MIP), semi‐invasive preparation (SIP), and invasive preparation (IP). After each preparation step, teeth were etched with 37% orthophosphoric acid for 15 s and subsequently evaluated using optical microscopy at 20× magnification and standardized postetching macrophotography. Enamel and dentin surfaces were quantified on both image sets using ImageJ software.

**Results:**

During MIP, no dentin was exposed. After SIP, dentin exposure averaged 22% ± 20% for central incisors and 22% ± 15% for lateral incisors (*p* = 0.93). After IP, dentin exposure increased to 84% ± 3% for central incisors and 69% ± 14% for lateral incisors (*p* = 0.0002). No significant differences were found between the methods in measuring the proportions of dentin.

**Conclusions:**

Postetching macrophotography provided dentin‐exposure measurements comparable to the reference optical microscopy method, supporting its use as a reliable and clinically applicable assessment tool. MIP and SIP preserved the majority of enamel in maxillary incisors, ensuring favorable bonding. A three‐stage classification system based on dentin exposure was developed to help practitioners adapt bonding protocols.

**Clinical significance:**

This classification system, combined with a method for assessing residual enamel, offers a guide for customizing bonding protocols in veneer preparation.

## 1. Introduction

Laminate veneers (LVs) have gained significant popularity in contemporary dentistry due to their durability, esthetic appeal [1], and minimal invasiveness [1–7]. These veneers are thin, custom‐fabricated, typically composed of ceramic or composite materials, that are bonded to the facial surface of teeth [8, 9].

Traditional veneers, typically 0.3–1.0 mm thick [10], have evolved toward more conservative approaches, including “minimal‐preparation” and “no‐preparation” techniques [11]. Minimal preparation veneers (MPVs), with a thickness of 0.2–0.5 mm [12, 13], aim to preserve dental structure and biological integrity, thereby reducing clinical complications [14]. Such conservative indications are particularly suitable in cases requiring limited ceramic application and may allow for future retreatment if necessary [15].

The clinical success (function and esthetics) and survival (in situ) of porcelain veneers depend on several factors, including the material used, marginal and internal fit [16], veneer thickness [17], fabrication method [16, 18], veneer location [5, 16], type of tooth preparation [4, 16], the underlying tooth substrate [7, 17, 19], the bonding technique [20] and the flexural strength of the restorative material [21].

Preserving tooth structure, particularly enamel, is crucial for successful veneer placement. A recent meta‐analysis by Alqtaibi et al. [22] reported that porcelain veneers bonded to enamel exhibit near‐perfect survival and success rates, ranging from 98% to 100%, whereas veneers bonded to composite resin or dentin‐exposed surfaces showed lower and more variable outcomes. Severe dentin exposure was associated with a marked reduction in both survival and success rates [22]. These clinical findings are consistent with long‐term survival data reported by Alenezi et al. [4] and with in vitro studies showing higher bond strength to enamel than to dentin, although such tests provide only indicative information and do not fully reproduce intraoral conditions [23]. When enamel retention falls below ~30%–40%, bonding predictability decreases, and protocols such as immediate dentin sealing may become necessary [24–27]. This underlines the importance of quantifying enamel and dentin exposure during veneer preparation.

Adequate bonding of porcelain veneers relies on accurately identifying dentin exposure during tooth preparation [28]. While significant dentin exposure may be necessary to address minor misalignments or discoloration [29], precise visualization remains crucial for an appropriate bonding strategy. Optical magnification systems enhance the visualization of dental hard tissues; however, variability in recognizing enamel and dentin among clinicians with different experience levels complicates this process [15, 28].

Several tools exist to differentiate enamel from dentin, but their clinical application during preparation presents challenges. X‐ray tomography provides precise visualization of enamel and dentin, but it is primarily a laboratory method [28, 30]. Other laboratory techniques include stereomicroscopy [1, 29] or microscopy and the use of dentin dyes [31] and chemical enamel dissolution using 5% formic acid [32].

Clinically, etching with agents like 37% phosphoric acid can distinguish between enamel and dentin by producing distinct surface morphologies and demineralization patterns [33]. Phosphoric acid etching for 30 s produces a chalky white enamel appearance, while shorter etching times (2–3 s) can create a glazed enamel and shiny dentin [34]. Gresnigt et al. [35] also used an etching gel, applied for 5 s, before taking pictures of the teeth and analyzing them with image processing software. Despite their potential, these methods lack validation against a definitive standard.

The introduction of postetching macrophotography offers a promising solution for predictable and clinically applicable control of tooth structure reduction during veneer preparation. Combined with digital analysis software [29], this approach can identify the areas and extent of tooth reduction, enhancing the precision and effectiveness of veneer placement.

This study aimed to compare the accuracy and reliability of postetching macrophotography, a clinically applicable method, with microscope observation, the laboratory reference method, for quantifying the surfaces of enamel and dentin. Additionally, it sought to assess the proportion of exposed dentin and enamel resulting from varying depths of veneer preparations and to develop a clinically relevant veneer classification based on surface characteristics (enamel vs. dentin) to optimize bonding protocols.

To our knowledge, this is the first study to validate a clinically applicable, noninvasive photographic method—postetching macrophotography—against a laboratory reference for quantifying enamel and dentin exposure. It also introduces a simplified, visual classification system for the prepared tooth surface, specifically designed to support clinical decision‐making based on tissue characteristics (enamel vs. dentin exposure). The null hypothesis was that postetching macrophotography would measure the same tissue‐exposure areas as the reference method, with enamel remaining the predominant tissue on the surface to receive the bonding protocol.

## 2. Materials and Methods

### 2.1. Study Design

**Figure 1:**
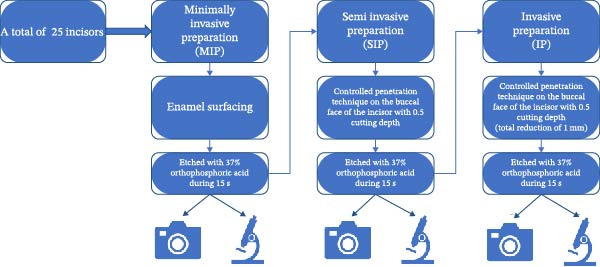
Flow chart of the study.

An ex vivo study was conducted at the Toulouse Health Faculty (Département d’Odontologie, Faculté de Santé de Toulouse, Université Toulouse III Paul Sabatier, Toulouse, France). As an ex vivo study, no clinical follow‐up was applicable. The reference method used to compare postetching macrophotography was microscopic observation (Figure 1).

### 2.2. Samples Preparation

The sample consisted of 25 human maxillary incisors (12 central and 13 lateral) selected to reflect anatomical variability across anterior teeth. The sample was collected in Toulouse in compliance with the hospital’s rules for tissue collection (DC‐2022‐5010). This sample size was based on previous ex vivo studies evaluating enamel preservation and dentin exposure during veneer preparation, which used between 10 and 36 anterior teeth for descriptive and comparative purposes [28, 29, 36]. The distribution allowed for representative assessments of different preparation depths while maintaining standardized conditions. All teeth were extracted for periodontal reasons and, with no restorations or signs of wear, were collected following patient authorization. Postextraction, the teeth underwent scaling and decontamination and then were preserved in a 1% chloramine solution for a period comprised between 1 week and 1 month before use. Each tooth was then thoroughly examined by visual inspection to confirm the absence of decay, fillings, or tissue loss. The roots of the teeth were embedded in standardized silicone boxes (Eurosil Max^2^ Putty, Henry Schein, Melville, NY, US) fabricated from a 3D‐printed mold to ensure precise repositioning during photographic documentation. Teeth were positioned in these molds with standardized alignment, free of rotation, malposition, or occlusal interference, to ensure consistent photographic and microscopic evaluation conditions. Additionally, a silicon key was designed for each tooth and used during the semi‐invasive preparation (SIP) and invasive preparation (IP) steps to help control and standardize the preparation depth throughout the study.

### 2.3. Tooth Preparation for Veneer Restoration

**Figure 2:**
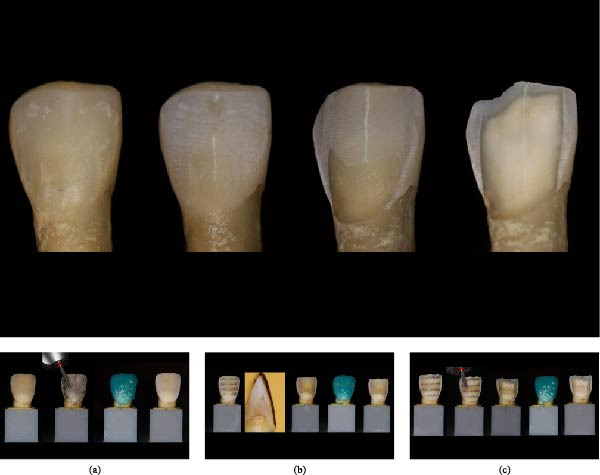
Representative maxillary central incisor illustrating the preparation sequence. From left to right: Initial tooth before etching, minimally invasive preparation (MIP) after etching, semi‐invasive preparation (SIP) after etching, and invasive preparation (IP) after etching. (a) Step 1—minimally invasive preparation (MIP). From left to right: Intact tooth before any preparation and before etching, buccal surface marked with graphite pencil to guide orientation prior to preparation (unetched), application of a red‐ring diamond bur to perform superficial enamel surfacing, tooth after etching with 37% orthophosphoric acid for 15 s, revealing enamel‐dentin contrast for analysis. (b) Step 2—semi‐invasive preparation (SIP). From left to right: Placement of calibrated horizontal index grooves using a 0.5 mm depth‐cutting bur, sectioned silicone key used to verify 0.5 mm penetration depth (buccolingual reference plane), SIP after completing 0.5 mm controlled reduction, tooth after etching with 37% orthophosphoric acid for 15 s, showing partial dentin exposure. (c) Step 3—invasive preparation (IP). From left to right: Placement of additional controlled‐depth grooves to achieve a total reduction of 1 mm, reduction continued following initial SIP, guided by depth grooves and silicone key verification, complete invasive preparation (1.0 mm total reduction), tooth after etching with 37% orthophosphoric acid for 15 s, illustrating predominant dentin exposure.

A single operator (AG), formally trained in prosthodontics and experienced in standardized veneer preparation protocols, performed all tooth preparations using 2.5x magnification loupes (Orascoptic, Kerr, Middleton, WI, USA). Each tooth underwent a specific three‐step preparation sequence [1], designed to transition from a highly conservative approach to a less conservative one (Figure 1), based on strict tissue economy principles:

• The first step, termed minimally invasive preparation (MIP), consisted of enamel surfacing with a red ring, fine‐grit tapered round‐end diamond bur (ISO: 806 314 198 514) (Stoner Dental, Toulouse, France) under air–water spray. To standardize the procedure, the buccal surface was marked with a graphite pencil, and reduction continued until the graphite was entirely removed (Figure 2a). This visual endpoint ensured minimal and consistent enamel reduction without altering tooth morphology.
• The second step, SIP, consisted of creating depth‐indicating grooves on the buccal surface using a calibrated depth‐cutting bur designed to achieve a reproducible 0.5 mm reduction (ISO 834.314.552.524.021; Stoner Dental, Toulouse, France). The grooves were then connected using a fine‐grit tapered round‐end diamond bur (ISO 806.314.198.514; Stoner Dental, Toulouse, France) under air–water spray to obtain a uniform preparation depth (Figure 2b).
• The third step, IP consisted of repeating the SIP protocol using the same instruments and parameters, resulting in a cumulative buccal reduction of ~1 mm (Figure 2c). The objective of the IP step was to simulate a clinically relevant deeper preparation that predictably results in substantial dentin exposure, enabling comparison of tissue quantification methods across distinct preparation depths.

The consistency of SIP and IP was maintained using a previously fabricated silicone key sectioned vertically in a buccolingual orientation. The key was positioned along the tooth’s widest buccolingual axis, contacting the incisal edge and buccal contour, to verify that the planned cumulative buccal reduction was achieved at several reference levels (cervical, middle, and incisal).

After each preparation step, a standardized sequence of procedures was applied:

• The tooth surfaces were polished with fine‐ and superfine‐grit Soft‐Lex polishing discs (3M Company, St. Paul, MN, USA) using progressively finer grits to remove debris and achieve a clean, uniform surface.
• The teeth were then etched with 37% orthophosphoric acid for 15 s, rinsed, and gently dried to allow clear visualization and photographic documentation of enamel and dentin boundaries for subsequent measurements, without influencing the preparation depth (Figure 2). The acid was applied for 15 s, followed by thorough rinsing and gentle air‐drying, comparable to clinical practice‐removing surface water without desiccating the tooth structure.
• Macrophotographic and microscopic images were acquired immediately after the etching procedure.
• A rehydration period of 30 min was observed before proceeding with each subsequent preparation step to allow the dentin to rehydrate before the next measurement step, ensuring consistent visual conditions across all observations. This rehydration phase was used solely for methodological standardization and is not part of clinical bonding protocols.

### 2.4. Microscopy Evaluation

After each preparation, etched teeth were examined under a 20x magnification optical microscope (Leica, Wetzlar, Germany). Microscopic observations were recorded as digital images using a dedicated EOS 700D camera (Canon Inc., Tokyo, Japan) mounted on the microscope. This method served as the reference for assessing dentin.

### 2.5. Macrophotography Evaluation

Following microscopic observation, standardized photographs of the buccal surfaces of the teeth were taken using a Canon EOS 700D camera (Canon Inc., Tokyo, Japan) equipped with a Canon Macro Lens EF 100 mm 1:2.8 USM and a Macro Ring Lite MR‐14 EX‐II flash (Canon Inc., Tokyo, Japan). Images were obtained under controlled lighting conditions and with a fixed camera angulation to ensure standardized imaging across samples.

### 2.6. Pictures Analysis

**Figure 3:**
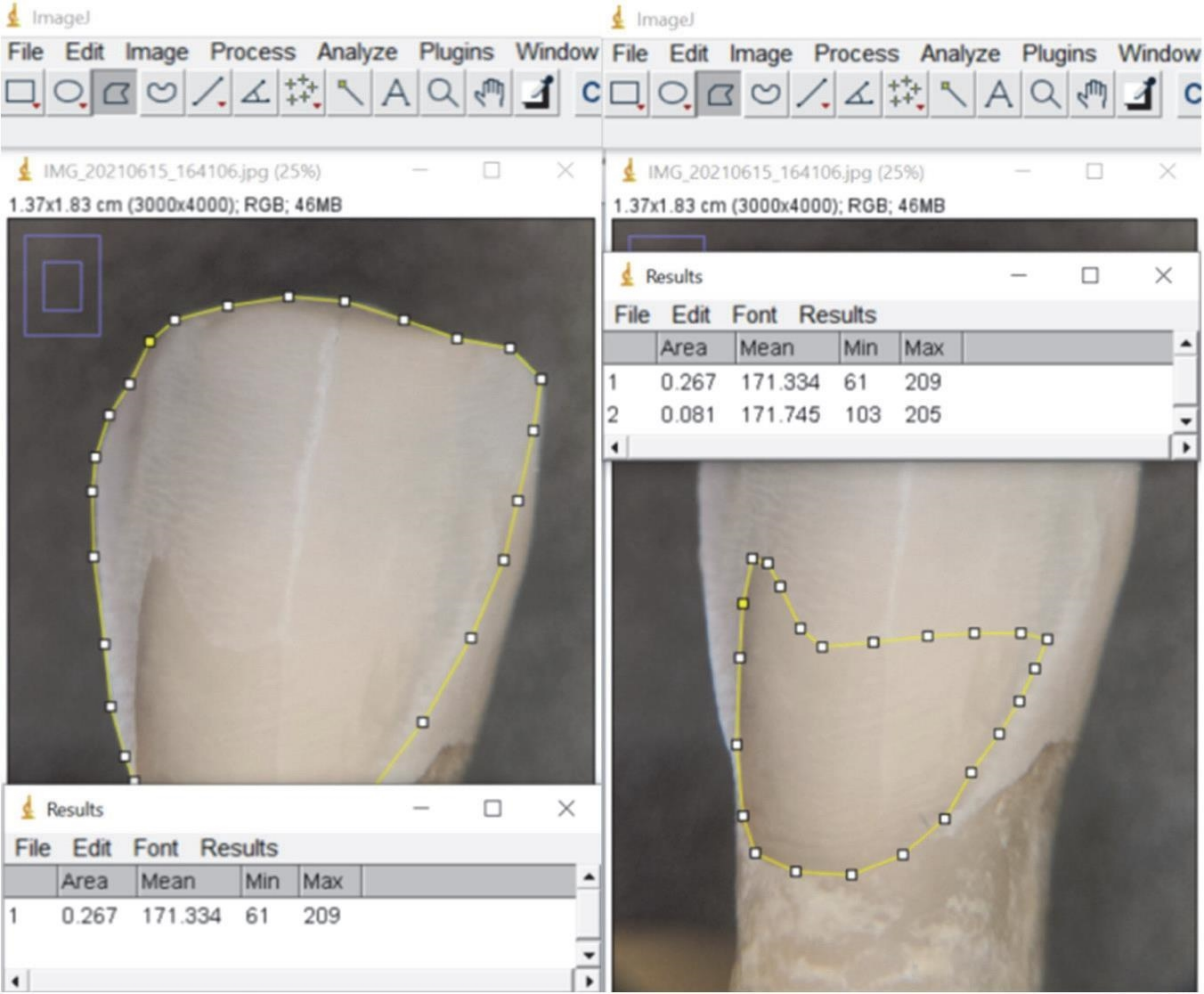
Microscopic evaluation of the prepared surface (reference method). Left: Total prepared surface outlined under 20x optical microscopy using ImageJ. Right: Dentin‐exposed area delineated on the same specimen to quantify the proportion of dentin within the prepared surface. Both measurements were performed on calibrated images using the polygon selection tool.

**Figure 4:**
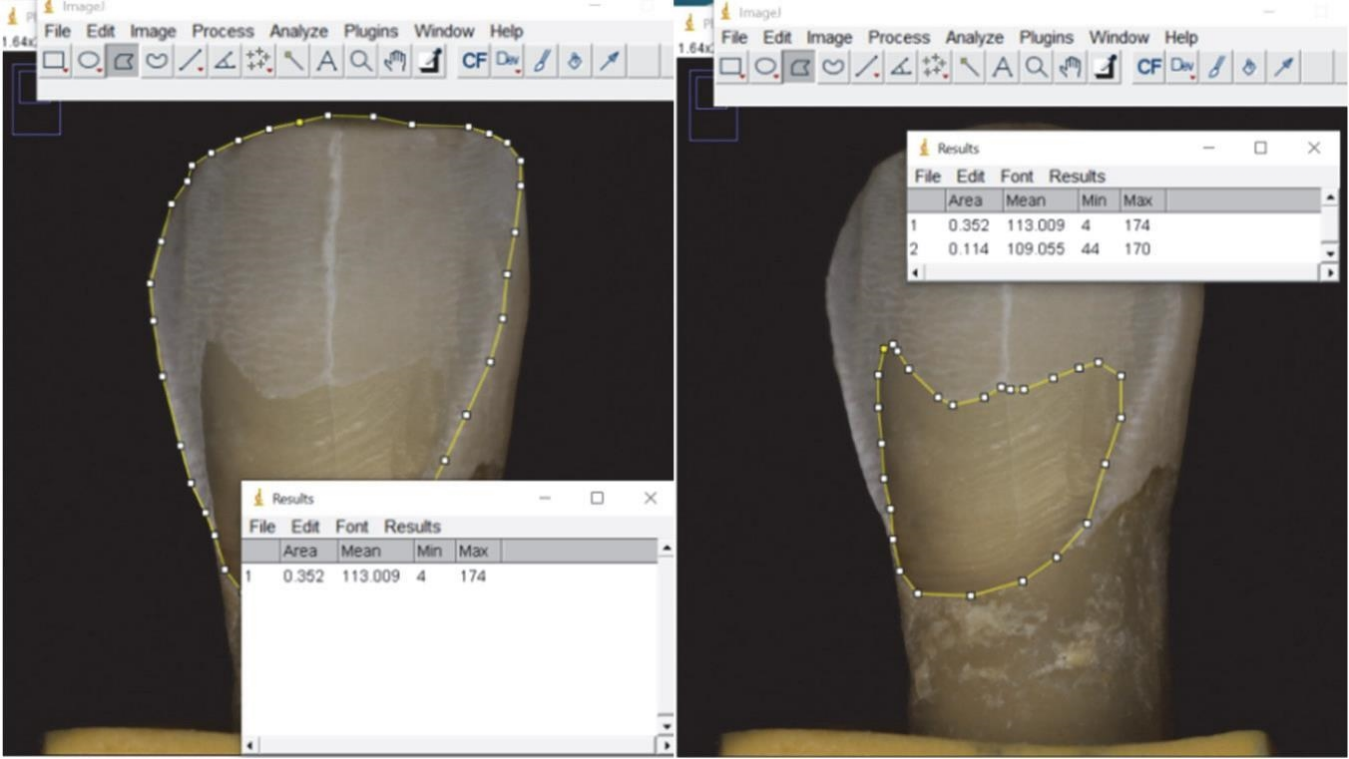
Evaluation of the total surface preparation and the dentin surface evaluation with macrophotography (tested method).

**Figure 5:**
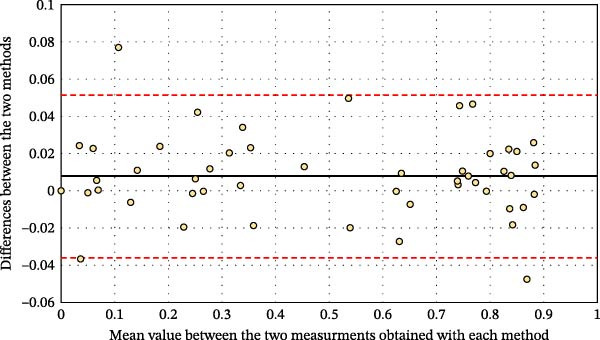
Bland–Altman diagram showing concordance between the two evaluation methods. The dark line indicates the mean difference between the microscopy and postetching macrophotography measurements. The two plotted red lines indicate the upper and lower limits of the concordance interval (±1.96 standard deviation). The plot shows, for each tooth, the difference between the two methods against their mean. The solid line represents the mean difference, and the red dashed lines indicate the 95% limits of agreement. Most values fall within these limits with minimal dispersion, indicating good agreement between microscopy and macrophotography.

Enamel and dentin surfaces were measured using ImageJ software (v. 1.53 k, Wayne Rasband, NIH, USA) on both images obtained from microscopic observations (Figure 3) and photographs taken with the macro lens (Figure 4). The standardized dimensions of the silicon mold enabled the calibration of the image scales. Then, the “polygon selections” tool within the software was used to delineate and measure the exposed tissues (Figures 4 and 5). Each measurement was performed twice, with a 1‐month interval, by two trained evaluators (MU and TC) to ensure consistency and reliability of the data.

### 2.7. Statistical Analysis

The study database was created using Microsoft Excel 2010 (Microsoft, WA, USA). Data analyses and graphical representations were conducted using Stata v.13 (StataCorp, TX, USA) and GraphPad Prism 5 (GraphPad, CA, USA).

Inter‐evaluator variability and intra‐evaluator reproducibility were assessed by calculating Kendall’s Tau and Pearson’s correlation coefficients for 10 randomly chosen measurements. Agreement between methods was further examined using Bland–Altman analysis to evaluate bias and limits of agreement. Group comparisons were performed using either Student’s or the Mann–Whitney Wilcoxon test, depending on whether the assumptions of normal distribution and variance equality were met. A 5% significance level was used for all statistical tests.

### 2.8. Tooth‐Surface Classification for Veneer Treatment Classification

Based on the enamel and dentin surface characteristics observed at different preparation depths, an exploratory tooth‐surface classification was proposed to help practitioners estimate the proportion of residual enamel after preparation.

## 3. Results

### 3.1. Samples

Among the 25 teeth, 12 were maxillary central incisors, and 13 were maxillary lateral incisors. The surface ratio (dentin/enamel) was measured for each degree of preparation and for each method of evaluation. This comprehensive approach ensured robust data collection across different tooth types and preparation stages, providing a thorough assessment of enamel and dentin exposure under varying conditions.

### 3.2. Method Validation

Inter‐evaluator variability was deemed excellent, with Kendall’s tau coefficients of 0.95 (*p*  < 0.0001) for the microscopy (M) method and 0.98 (*p*  < 0.0001) for the etching (E) method. Intra‐evaluator reproducibility was also outstanding, as indicated by Pearson’s correlation coefficient of 0.97.

Regardless of the type of preparation, there was no significant difference in the proportion of dentin measured between the two methods (Table 1). This finding was corroborated by the excellent concordance observed in the Bland–Altman plot (Figure 5).

**Table 1:** Comparison between dentin exposure measured on microscopy and by postetching macrophotography (*n* = 25 observations for each case).

Type of preparation	Microscopy (%)	Photo after etching (%)	*p*
MIP	0	0	>0.99
SIP	21 ± 17	22 ± 17	0.76
IP	76 ± 12	77 ± 12	0.78

*Note:* Values represent the mean percentage of the prepared buccal surface identified as dentin (mean ± SD). A value of 0% indicates no dentin exposure. The *p*‐values correspond to paired comparisons between microscopy and macrophotography for each preparation stage.

Abbreviations: IP, invasive preparation; MIP, minimally invasive preparation; SIP, semi‐invasive preparation.

### 3.3. Dentin Exposure

Dentin was never exposed during MIP. However, the percentage of dentin surface increased following SIP and IP. For SIP, the dentin exposure averaged 22% ± 20% for central incisors and 22% ± 15% for lateral incisors (*p* = 0.93). In contrast, IP resulted in 84% ± 3% of the surface being composed of dentin for central incisors and 69% ± 14% for lateral incisors (*p* = 0.0002).

In all instances, dentin exposure was predominantly located at the cervical regions of the teeth and did not affect the peripheral band, which remained entirely composed of enamel (Figure 1). This consistent pattern underscores the importance of preparation depth in influencing dentin exposure, particularly in critical areas for bonding and veneer placement.

### 3.4. Veneer Classification

Based on the data obtained in this study, we developed an exploratory visual classification intended for clinical use during tooth preparation for veneer bonding. This classification system comprises three distinct stages (Figure 6):

Figure 6Classification of residual enamel after veneer preparation. (a) Stage 1—complete enamel preservation, with no dentin exposure. (b) Stage 2: partial dentin exposure (<50% of the buccal surface), typically located centrally and/or cervically, with enamel remaining mainly on the incisal and proximal margins rather than as a continuous cervical rim. (c) Stage 3: predominant dentin exposure (>50%), with residual enamel limited to small peripheral segments.

**Figure:**
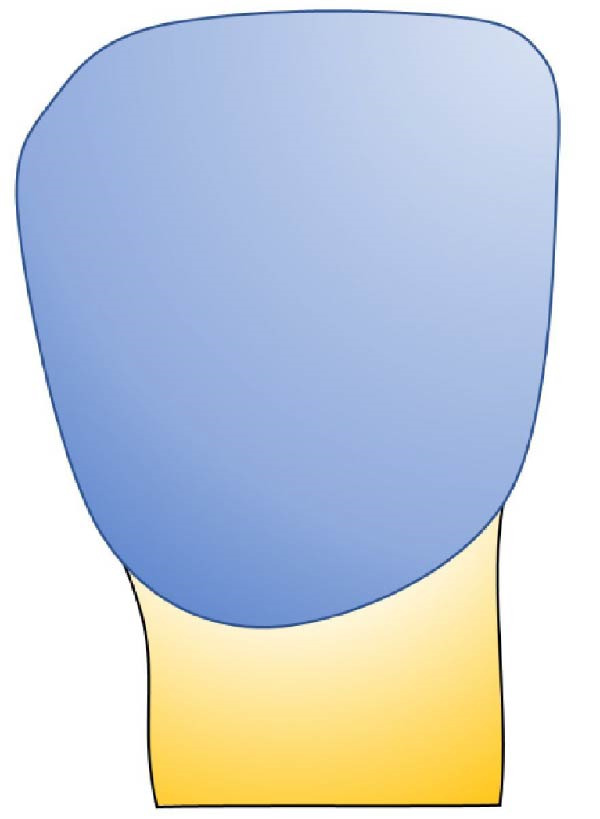
(a)

**Figure:**
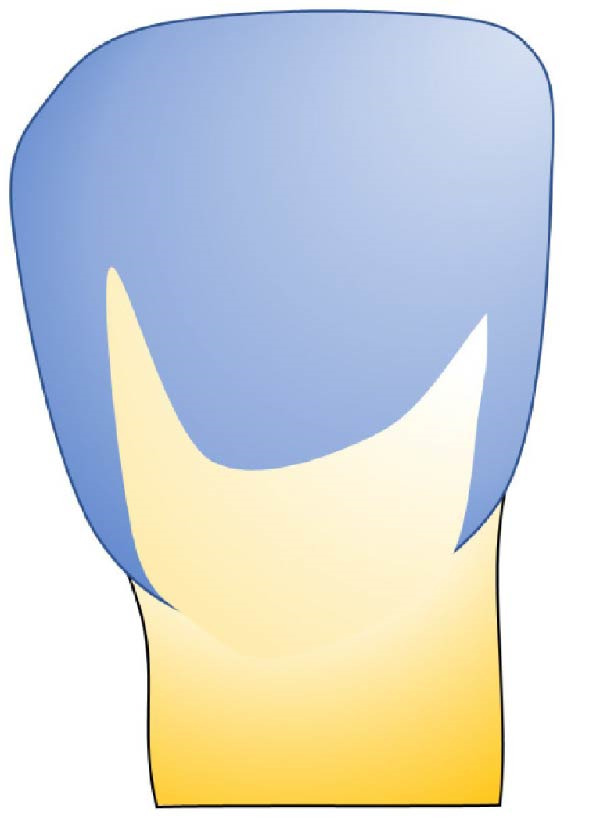
(b)

**Figure:**
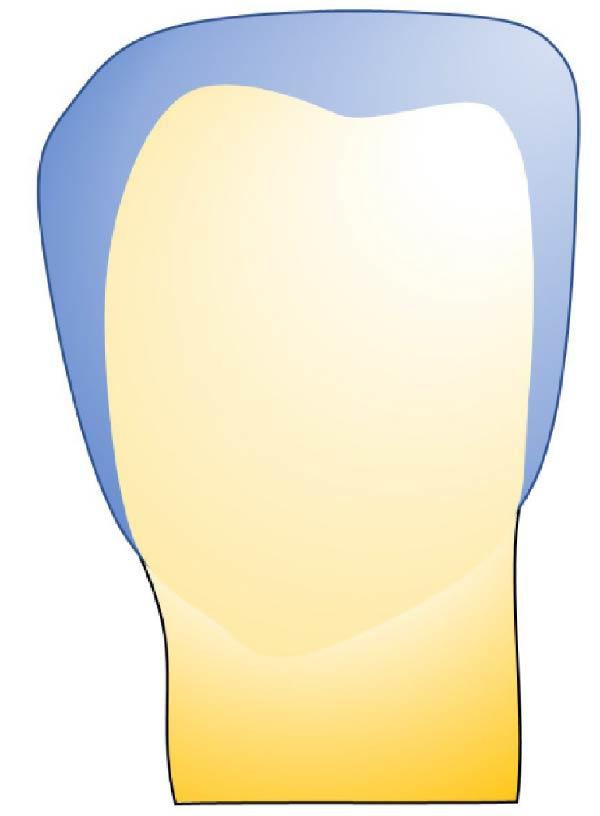
(c)

• Stage 1: no dentin exposure on the prepared surface.
• Stage 2: the dentin surface occupies less than half of the prepared surface.
• Stage 3: predominant dentin area surrounded by a peripheral enamel frame.

This classification provides a clear, visual guide for clinicians, facilitating precise assessment and decision‐making for the bonding procedure during veneer treatment. By categorizing the extent of dentin exposure, this system is intended to better guide clinicians in selecting appropriate bonding strategies and to facilitate standardized reporting of preparation conditions in future research.

## 4. Discussion

This study evaluated the efficacy and reliability of postetching macrophotography as a clinical tool for quantifying enamel and dentin surfaces, compared to the reference method of microscopic observation. The results demonstrate that postetching macrophotography is a reliable and clinically applicable technique, offering the advantage of immediate feedback during veneer preparation, making it well‐suited for real‐time clinical settings.

### 4.1. Method Validation

Both methods—postetching macrophotography and microscopy—showed excellent interobserver consistency and intraobserver repeatability, confirming their reliability for measuring enamel and dentin exposure. The Bland–Altman analysis further validated the precision of macrophotography, showing no significant discrepancies between the two methods. This validation supports its use as an alternative to laboratory‐based microscopy in clinical practice.

### 4.2. Clinical Relevance of Postetching Macrophotography

Because phosphoric acid etching was systematically performed prior to analysis in both methods, enamel and dentin surfaces exhibited comparable etching‐related optical contrasts regardless of whether evaluation was conducted using optical microscopy or macrophotography. In addition, similar magnification and standardized flash conditions contributed to consistent visualization of the etched surfaces. These methodological similarities likely explain why both approaches yielded comparable quantitative measurements in this study. An essential advantage of it is that it can be performed directly in the clinical setting without relying on laboratory‐based or destructive techniques such as crown sectioning, which are limited to in vitro investigations [29, 36]. The method also demonstrates strong reproducibility across examiners. Furthermore, as noted by Sorrentino et al. [28], the analysis and identification of dentin become more challenging as the extent of the exposed surface increases. These features highlight the potential of postetching macrophotography as a practical and accessible tool for clinicians and for training purposes, facilitating the distinction between enamel and dentin during veneer preparation.

In our study, a 15 s application of 37% phosphoric acid was used across all samples. This duration corresponds to the minimum generally recommended for dentin, as opposed to the 30 s typically applied to enamel [37]. The aim was to determine whether this conservative etching time would still provide sufficient tissue contrast for macrophotographic differentiation. Although longer etching may enhance enamel contrast, our protocol was chosen to minimize over‐demineralization and maintain clinical relevance. Moreover, using the same etching time across all samples improved measurement reproducibility. Previous studies have shown that etching time affects the morphology of both enamel and dentin surfaces [37, 38], which may influence reflectivity and contrast under standardized photographic conditions, thereby supporting our methodological approach.

### 4.3. Enamel Exposure and Preservation

In this study, MIP and SIP were predominantly intra‐enamel in nature on maxillary incisors. This is consistent with prior findings that emphasize the potential for achieving complete intra‐enamel preparation when minimally invasive tooth preparation techniques are employed, especially in cases where no palatal reduction is required [36].

Notably, software analysis revealed that while most labial reductions in SIP occurred within the enamel, areas such as the cervical third tended to expose dentin due to the natural tapering of enamel thickness near the cementoenamel junction (CEJ) [39, 40].

Adhesive protocols benefit greatly from retaining at least 40% of enamel to ensure long‐lasting bonding strength [24, 25]. This approach reinforces adherence to the therapeutic gradient, advocating minimal intervention whenever possible before considering more invasive options.

### 4.4. Dentin Exposure and Preservation

The cervical predominance of dentin exposure observed in this study is consistent with previous findings. Cherukara et al. [39] reported that even with depth‐calibrated reduction of 0.4–0.6 mm, enamel thinning toward the CEJ frequently results in cervical dentin exposure, a pattern also described by Nattress et al. [31]. After IP, there was a significant difference between tooth types, with central incisors exhibiting more dentin exposure than laterals. Because the actual reduction depth for each stage (MIP, SIP, and IP) was not directly measured, slight variations may have contributed to this difference. Although lateral incisors are generally described as having thinner enamel overall, Gao et al. [36] reported that enamel distribution differs between tooth types, with laterals retaining proportionally more midfacial enamel at comparable reduction depths (0.6–0.7 mm) [36]. This anatomical variation may help explain the lower dentin exposure seen in lateral incisors and underscores the importance of considering enamel morphology when planning deeper preparations.

### 4.5. Veneers Classification

This study introduces an exploratory three‐stage visual classification system based on the extent and pattern of enamel and dentin exposure after veneer preparation. Although not assessed in this study, previous research suggests that adapting bonding strategies to the exposed substrate may influence adhesive performance [41, 42].

The proposed system simplifies existing frameworks, such as the classification by Le Sage [43], by focusing primarily on residual enamel.

• Stage 1: No dentin exposure, with bonding performed purely on enamel, ensuring optimal success and survival rates [41].
• Stage 2: Dentin exposure covering less than 50% of the prepared surface, typically located in the central and cervical areas while the peripheral enamel band remains intact.
• IDS is recommended to protect the exposed dentin, with no significant difference in survival rates compared to Stage 1 [41, 42].
• Stage 3: Majority dentin exposure surrounded by a peripheral enamel frame. IDS is also necessary at this stage, though survival rates may be lower compared to bonding predominantly on enamel [41, 42].

This simplified classification (Figure 6) provides clinicians with a clear, actionable framework for selecting bonding protocols, thereby improving bond strength and durability for LVs.

### 4.6. Impact of Etching Protocols on Bonding Efficacy

The veneer bonding protocol typically begins with tooth preparation, followed by enamel etching—unless significant dentin exposure requires additional considerations. In this context, phosphoric acid etching should be considered primarily as a diagnostic aid rather than a definitive bonding step, allowing accurate visual discrimination between enamel and dentin. This approach enables a strictly selective application of IDS, which should be limited to exposed dentin surfaces in accordance with established clinical protocols [20, 34]. When diagnostic etching is followed by temporization, the initially etched enamel surface does not represent the final bonding substrate. Prior to definitive veneer insertion, enamel can be safely reconditioned, for instance by gentle air‐abrasion to remove contaminants, and selectively re‐etched to restore an optimal etching pattern without compromising adhesion [41]. In contrast, IDS‐treated dentin should not be re‐etched, as its reactivation relies on mechanical surface treatment rather than additional phosphoric acid application. While phosphoric acid etching helps differentiate enamel from dentin, its use for visual assessment raises concerns about its impact on bonding efficacy.

Over‐etching enamel or dentin with 37% phosphoric acid can lead to excessive demineralization, increased surface roughness, and reduced enamel microhardness, all of which may compromise the integrity of the hybrid layer and long‐term bond stability [26, 41]. In some instances, excessive etching has also been linked to increased nanoleakage and inadequate resin infiltration [44]. Although certain studies suggest that even 60 s of etching can maintain acceptable bond strength values [45], others emphasize that durations exceeding 30 s do not significantly improve performance and may instead increase risk. These inconsistencies underscore the need for standardized etching protocols.

### 4.7. Strengths and Limitations

The main strength of this study lies in the validation of postetching macrophotography as a clinically relevant method for quantifying enamel and dentin surfaces.

A single trained operator conducted all preparations, a decision based on both convenience and on eliminating interoperator variability, an important factor in validation studies.

Although blinding was not feasible for the evaluators due to the visible differences between preparation stages, potential bias was minimized through standardized assessment procedures, and reproducibility was confirmed by strong inter‐ and intra‐evaluator agreement. The sample size of 25 teeth, consistent with previous ex vivo studies, was sufficient for controlled comparative assessments and methodological validation [28, 29, 36]. While not intended to represent population‐wide variability, the standardized design enabled reproducible measurements and robust internal comparisons.

Several limitations must be acknowledged. The in vitro design does not replicate intraoral conditions such as saliva, temperature, humidity, patient movement, or occlusal forces, which may influence adhesive behavior [46–48]. Moreover, because clinical photography is subject to variability in lighting and angulation [49], the external validity of our imaging‐based measurements may be limited.

The study also focused solely on the buccal surface, without addressing palatal or proximal preparations.

A limitation of this study is that actual reduction depths for MIP, SIP, and IP were not directly measured. Although preparation depth was controlled with calibrated depth‐cutting burs and verified with a sectioned silicone key along a buccolingual plane, small localized variations outside this reference plane cannot be excluded. These variations may have influenced the extent of dentin exposure. Future studies incorporating multiplane or volumetric assessment methods, such as three‐dimensional scanning, could provide a more comprehensive evaluation of preparation uniformity.

These methodological insights provide a foundation for future in vivo validation and broader clinical integration.

### 4.8. Perspectives and Clinical Implications

Future studies should evaluate this approach in clinical settings, incorporate multiple operators to assess interoperator variability, and explore more complex preparation designs to confirm the reproducibility of our findings. Digital tools such as intraoral scanning and 3D surface analysis may also help quantify enamel and dentin exposure and could complement postetching macrophotography preservation.

## 5. Conclusions

This study demonstrates the reliability of postetching macrophotography in accurately distinguishing between enamel and dentin during veneer preparation. The proposed three‐stage classification system provides clinicians with a structured approach to tailor bonding protocols based on the degree of enamel preservation. The study findings indicate that after a 0.5 mm tissue reduction on the buccal surface of maxillary incisors, the percentage of exposed dentin remains well below 50%, ensuring that adequate enamel is preserved for optimal bonding.

## Author Contributions

Conceptualization: Antoine Galibourg. Project administration: Antoine Galibourg. Investigation: Manon Udar. Data curation: Manon Udar. Formal analysis: Thibault Canceill and Constance Cuny. Writing – original draft preparation: Constance Cuny and Thibault Canceill. Writing – review and editing: Constance Cuny, Markus B. Blatz, and Antoine Galibourg. Visualization: Constance Cuny. Resources: Thibault Canceill. Supervision: Markus B. Blatz.

## Funding

The authors have nothing to report.

## Disclosure

All authors have read and agreed to the published version of the manuscript.

## Ethics Statement

The authors have nothing to report.

## Conflicts of Interest

The authors declare no conflicts of interest.

## Data Availability

The data that support the findings of this study are available from the corresponding author upon reasonable request.

## References

[1] Blunck U. , Fischer S. , Hajtó J. , Frei S. , and Frankenberger R. , Ceramic Laminate Veneers: Effect of Preparation Design and Ceramic Thickness on Fracture Resistance and Marginal Quality In Vitro, Clinical Oral Investigations. (2020) 24, no. 8, 2745–2754, 10.1007/s00784-019-03136-z. 31900673

[2] Alothman Y. and Bamasoud M. S. , The Success of Dental Veneers According To Preparation Design and Material Type, Open Access Macedonian Journal of Medical Sciences. (2018) 6, no. 12, 2402–2408, 10.3889/oamjms.2018.353, 2-s2.0-85061097543. 30607201 PMC6311473

[3] Liu M. , Gai K. , Chen J. , and Jiang L. , Comparison of Failure and Complication Risks of Porcelain Laminate and Indirect Resin Veneer Restorations: A Meta-Analysis, The International Journal of Prosthodontics. (2018) 32, no. 1, 59–65, 10.11607/ijp.6099, 2-s2.0-85060516475. 30677113

[4] Alenezi A. , Alsweed M. , Alsidrani S. , and Chrcanovic B. R. , Long-Term Survival and Complication Rates of Porcelain Laminate Veneers in Clinical Studies: A Systematic Review, Journal of Clinical Medicine. (2021) 10, no. 5, 10.3390/jcm10051074, 1074. 33807504 PMC7961608

[5] Rinke S. , Bettenhäuser-Hartung L. , Leha A. , Rödiger M. , Schmalz G. , and Ziebolz D. , Retrospective Evaluation of Extended Glass-Ceramic Ceramic Laminate Veneers After a Mean Observational Period of 10 Years, Journal of Esthetic and Restorative Dentistry. (2020) 32, no. 5, 487–495, 10.1111/jerd.12597. 32452164

[6] Arif R. , Dennison J. B. , Garcia D. , and Yaman P. , Retrospective Evaluation of the Clinical Performance and Longevity of Porcelain Laminate Veneers 7 to 14 Years After Cementation, The Journal of Prosthetic Dentistry. (2019) 122, no. 1, 31–37, 10.1016/j.prosdent.2018.09.007, 2-s2.0-85063026789. 30885576

[7] Morimoto S. , Albanesi R. B. , Sesma N. , Agra C. M. , and Braga M. M. , Main Clinical Outcomes of Feldspathic Porcelain and Glass-Ceramic Laminate Veneers: A Systematic Review and Meta-Analysis of Survival and Complication Rates, The International Journal of Prosthodontics. (2016) 29, no. 1, 38–49, 10.11607/ijp.4315. 26757327

[8] El-Mowafy O. , El-Aawar N. , and El-Mowafy N. , Porcelain Veneers: An Update, Dental and Medical Problems. (2018) 55, no. 2, 207–211, 10.17219/dmp/90729, 2-s2.0-85049156852. 30152626

[9] The Glossary of Prosthodontic Terms, Journal of Prosthetic Dentistry. (2017) 117, no. 5, C1–e105. 10.1016/j.prosdent.2016.12.001 28418832

[10] Meer Rownaq Ali A. , Conventional Versus Minimally Invasive Veneers: A Systematic Review, Cureus. (2023) 15, no. 9, 10.7759/cureus.44638, e44638. 37799216 PMC10548404

[11] Gürel G. , Porcelain Laminate Veneers: Minimal Tooth Preparation by Design, Dental Clinics of North America. (2007) 51, no. 2, 419–431, ix 10.1016/j.cden.2007.03.007, 2-s2.0-34249692426. 17532920

[12] Gresnigt M. and Ozcan M. , Esthetic Rehabilitation of Anterior Teeth With Porcelain Laminates and Sectional Veneers, Journal of the Canadian Dental Association. (2011) 77, b143. 22067068

[13] Radz G. M. , Minimum Thickness Anterior Porcelain Restorations, Dental Clinics of North America. (2011) 55, no. 2, 353–370, 10.1016/j.cden.2011.01.006, 2-s2.0-79953320015. 21473998

[14] Ciora E. , Miron M. , Bojoga D. , Lungeanu D. , and Jivanescu A. , Evaluation of the Pulp Chamber Temperature During Tooth Veneer Preparation Using Burs With Different Degrees of Wear—A Preliminary In Vitro Study, Dentistry Journal. (2023) 11, no. 8, 10.3390/dj11080197, 197. 37623293 PMC10453045

[15] Robles M. , Jurado C. A. , Azpiazu-Flores F. X. , Villalobos-Tinoco J. , Afrashtehfar K. I. , and Fischer N. G. , An Innovative 3D Printed Tooth Reduction Guide for Precise Dental Ceramic Veneers, Journal of Functional Biomaterials. (2023) 14, no. 4, 10.3390/jfb14040216, 216. 37103306 PMC10146615

[16] Baig M. R. , Qasim S. S. B. , and Baskaradoss J. K. , Marginal and Internal Fit of Porcelain Laminate Veneers: A Systematic Review and Meta-Analysis, The Journal of Prosthetic Dentistry. (2024) 131, no. 1, 13–24, 10.1016/j.prosdent.2022.01.009. 35260253

[17] Ge C. , Green C. C. , Sederstrom D. A. , McLaren E. A. , Chalfant J. A. , and White S. N. , Effect of Tooth Substrate and Porcelain Thickness on Porcelain Veneer Failure Loads In Vitro, The Journal of Prosthetic Dentistry. (2018) 120, no. 1, 85–91, 10.1016/j.prosdent.2017.10.018, 2-s2.0-85038847431. 29273236

[18] Sen N. and Olley R. C. , Retrospective Evaluation of Factors Affecting Long-Term Clinical Performance of CAD-CAM Laminate Veneers, International Journal of Prosthodontics. (2023) 37, no. 4, 411–416. 10.11607/ijp.8499 37824337

[19] Burke F. J. T. , Survival Rates for Porcelain Laminate Veneers With Special Reference to the Effect of Preparation in Dentin: A Literature Review, Journal of Esthetic and Restorative Dentistry. (2012) 24, no. 4, 257–265, 10.1111/j.1708-8240.2012.00517.x, 2-s2.0-84864695265. 22863131

[20] Gresnigt M. M. M. , Cune M. S. , and Schuitemaker J. , et al.Performance of Ceramic Laminate Veneers With Immediate Dentine Sealing: An 11 Year Prospective Clinical Trial, Dental Materials. (2019) 35, no. 7, 1042–1052. 31084936 10.1016/j.dental.2019.04.008

[21] Munoz A. , Zhao Z. , Paolone G. , Louca C. , and Vichi A. , Flexural Strength of CAD/CAM Lithium-Based Silicate Glass–Ceramics: A Narrative Review, Materials. (2023) 16, no. 12, 10.3390/ma16124398, 4398. 37374581 PMC10301111

[22] Alqutaibi A. Y. , Saker S. , Alghauli M. A. , Algabri R. S. , and AbdElaziz M. H. , Clinical Survival and Complication Rate of Ceramic Veneers Bonded to Different Substrates: A Systematic Review and Meta-Analysis, The Journal of Prosthetic Dentistry. (2025) 134, 1030–1039. 38604905 10.1016/j.prosdent.2024.03.019

[23] Öztürk E. , Bolay Ş. , Hickel R. , and Ilie N. , Shear Bond Strength of Porcelain Laminate Veneers to Enamel, Dentine and Enamel-Dentine Complex Bonded With Different Adhesive Luting Systems, Journal of Dentistry. (2013) 41, no. 2, 97–105, 10.1016/j.jdent.2012.04.005, 2-s2.0-84873716313. 22521701

[24] Zhu J. , Gao J. , Jia L. , Tan X. , Xie C. , and Yu H. , Shear Bond Strength of Ceramic Laminate Veneers to Finishing Surfaces With Different Percentages of Preserved Enamel Under a Digital Guided Method, BMC Oral Health. (2022) 22, no. 1, 10.1186/s12903-021-02038-5, 3. 34996438 PMC8742459

[25] Gresnigt M. M. M. , Braeckmans A. , van der Made S. A. M. , and Naves L. Z. , Partial Anterior Indirect Restorations in Cases With Dentin Exposure, The international journal of esthetic dentistry. (2021) 16, no. 4, 554–569. 34694079

[26] Ozer F. , Batu Eken Z. , Hao J. , Tuloglu N. , and Blatz M. B. , Effect of Immediate Dentin Sealing on the Bonding Performance of Indirect Restorations: A Systematic Review, Biomimetics. (2024) 9, no. 3, 10.3390/biomimetics9030182, 182. 38534867 PMC10968373

[27] Samimi P. , Iranmanesh P. , Khoroushi M. , Kafi M. H. , and Jafari N. , Bond Strength Evaluation of Ceramic Restorations With Immediate Dentin Sealing: A Systematic Review and Meta-Analysis, Journal of Dentistry. (2024) 25, no. 3, 192–202. 39371953 10.30476/dentjods.2023.97057.1986 PMC11452600

[28] Sorrentino R. , Ruggiero G. , Borelli B. , Barlattani A. , and Zarone F. , Dentin Exposure After Tooth Preparation for Laminate Veneers: A Microscopical Analysis to Evaluate the Influence of Operators’ Expertise, Materials. (2022) 15, no. 5, 1763. 35268994 10.3390/ma15051763 PMC8911512

[29] Pahlevan A. , Mirzaee M. , and Yassine E. , et al.Enamel Thickness After Preparation of Tooth for Porcelain Laminate, Journal of Dentistry. (2014) 11, no. 4, 428–432. 25584054 PMC4283744

[30] Wang P. , Sun F. , Yu Q. , and Wu G. , Three-Dimensional Analysis of the Relationship Between the Structure of Maxillary Central Incisor and the Preparation of Dental All-Ceramic, PLoS ONE. (2018) 13, no. 12, 10.1371/journal.pone.0209791, 2-s2.0-85059244977, e0209791. 30589894 PMC6307721

[31] Nattress B. R. , Youngson C. C. , Patterson C. J. , Martin D. M. , and Ralph J. P. , An In Vitro Assessment of Tooth Preparation for Porcelain Veneer Restorations, Journal of Dentistry. (1995) 23, no. 3, 165–170, 10.1016/0300-5712(95)93574-L, 2-s2.0-0029317685. 7782528

[32] Atria P. J. , Barbosa J. M. , Sampaio C. S. , Jorquera G. , Hirata R. , and Mahn G. , Comparison of a Non-Destructive Technique Using Three-Dimensional Imaging and Histoanatomical Chemical Dissolution for Dental Morphology Analysis, The international journal of esthetic dentistry. (2019) 14, no. 1, 76–85. 30714056

[33] Darzé F. M. , Bridi E. C. , França F. M. G. , do Amaral F. L. B. , Turssi C. P. , and Basting R. T. , Enamel and Dentin Etching With Glycolic, Ferulic, and Phosphoric Acids: Demineralization Pattern, Surface Microhardness, and Bond Strength Stability, Operative Dentistry. (2023) 48, no. 2, E35–E47, 10.2341/21-143-L. 36656318

[34] Magne P. , Immediate Dentin Sealing: A Fundamental Procedure for Indirect Bonded Restorations, Journal of Esthetic and Restorative Dentistry. (2005) 17, no. 3, 144–154, 10.1111/j.1708-8240.2005.tb00103.x. 15996383

[35] Gresnigt M. M. M. , Cune M. S. , de Roos J. G. , and Özcan M. , Effect of Immediate and Delayed Dentin Sealing on the Fracture Strength, Failure Type and Weilbull Characteristics of LithiumSdisilicate Laminate Veneers, Dental Materials. (2016) 32, no. 4, e73–81. 26856454 10.1016/j.dental.2016.01.001

[36] Gao J. , Jia L. , Tan X. , and Yu H. , Three-Dimensional Quantification of Enamel Preservation in Tooth Preparation for Porcelain Laminate Veneers: A Fully Digital Workflow In Vitro Study, Operative Dentistry. (2022) 47, no. 2, 183–189, 10.2341/20-286-L. 35029681

[37] Bolaños-Carmona V. , González-López S. , Briones-Luján T. , De Haro-Muñoz C. , and de la Macorra J. C. , Effects of Etching Time of Primary Dentin on Interface Morphology and Microtensile Bond Strength, Dental Materials. (2006) 22, no. 12, 1121–1129. 16388845 10.1016/j.dental.2005.09.008

[38] Ibrahim I. M. , Elkassas D. W. , and Yousry M. M. , Effect of EDTA and Phosphoric Acid Pretreatment on the Bonding Effectiveness of Self-Etch Adhesives to Ground Enamel, European Journal of Dentistry. (2019) 04, no. 4, 418–428, 10.1055/s-0039-1697862. PMC2948749 20922162

[39] Cherukara G. , Davis G. , Seymour K. , Zou L. , and Samarawickrama D. , Dentin Exposure in Tooth Preparations for Porcelain Veneers: A Pilot Study, The Journal of Prosthetic Dentistry. (2005) 94, no. 5, 414–420, 10.1016/j.prosdent.2005.08.016, 2-s2.0-27744581738. 16275300

[40] Ferrari M. , Patroni S. , and Balleri P. , Measurement of Enamel Thickness in Relation to Reduction for Etched Laminate Veneers, The International journal of periodontics & restorative dentistry. (1992) 12, no. 5, 407–413. 1343012

[41] Araujo E. and Perdigão J. , Anterior Veneer Restorations - An Evidence-Based Minimal-Intervention Perspective, The Journal of Adhesive Dentistry. (2021) 23, no. 2, 91–110. 33825424 10.3290/j.jad.b1079529

[42] Oztürk E. and Bolay S. , Survival of Porcelain Laminate Veneers With Different Degrees of Dentin Exposure: 2-Year Clinical Results, The Journal of Adhesive Dentistry. (2014) 16, no. 5, 481–489. 25279393 10.3290/j.jad.a32828

[43] LeSage B. , Establishing a Classification System and Criteria for Veneer Preparations, Compendium of Continuing Education in Dentistry. (2013) 34, no. 2, 104–114. 23556319

[44] Burrer P. , Dang H. , Par M. , Attin T. , and Tauböck T. T. , Effect of Over-Etching and Prolonged Application Time of a Universal Adhesive on Dentin Bond Strength, Polymers. (2020) 12, no. 12, 10.3390/polym12122902, 2902. 33287394 PMC7761786

[45] Gindri L. , Fröhlich T. , Rosso C. , and Rocha R. , Etching Time and Bonding of Adhesive Systems to Dentin of Primary Teeth: A Systematic Review and Meta-Analysis, International Journal of Paediatric Dentistry. (2021) 31, no. 1, 122–130, 10.1111/ipd.12711. 33405356

[46] Jacquot B. , Durand J. C. , Farge P. , Valcarcel J. , Deville de Périère D. , and Cuisinier F. , Influence of Temperature and Relative Humidity on Dentin and Enamel Bonding: A Critical Review of the Literature. Part 1. Laboratory Studies, The Journal of Adhesive Dentistry. (2012) 14, no. 5, 433–446. 23082311 10.3290/j.jad.a28388

[47] Stoleriu S. , Iovan G. , Nica I. , Pancu G. , and Adrian S. , The Influence of Saliva Contamination on Universal Adhesive Bonding to Enamel and Dentin, Stomatology Edu Journal. (2019) 6, no. 4, 230–236, 10.25241/stomaeduj.2019.6(4).art.2.

[48] Saraiva L. O. , Aguiar T. R. , Costa L. , Cavalcanti A. N. , Giannini M. , and Mathias P. , Influence of Intraoral Temperature and Relative Humidity on the Dentin Bond Strength: an In Situ Study, Journal of Esthetic and Restorative Dentistry. (2015) 27, no. 2, 92–99. 24629068 10.1111/jerd.12098

[49] Moussa C. , Hardan L. , and Kassis C. , et al.Accuracy of Dental Photography: Professional Vs. Smartphone’s Camera, BioMed Research International. (2021) 2021, 3910291. 34957302 10.1155/2021/3910291 PMC8694966

